# Airway and Systemic Immunoglobulin Profiling and Immune Response in Adult Asthma

**DOI:** 10.1007/s00408-024-00699-x

**Published:** 2024-05-07

**Authors:** Laura J. Walsh, Ashley Sullivan, Chris Ward, Eoin B. Hunt, Susan Lapthorne, Joseph A. Eustace, Liam J. Fanning, Barry J. Plant, Paul M. O’Byrne, John A. MacSharry, Desmond M. Murphy

**Affiliations:** 1 The School of Medicine, University College Cork, Cork, Ireland; 2 The Department of Respiratory Medicine, Cork University Hospital, Cork, Ireland; 3 The HRB funded Clinical Research Facility, University College Cork, Cork, Ireland; 4 APC Microbiome Ireland, University College Cork, Cork, Ireland; 5 The School of Microbiology, University College Cork, Cork, Ireland; 6 Translational and Clinical Research Institute Cellular Medicine, Newcastle University, Newcastle upon Tyne, UK; 7 The Michael G DeGroote School of Medicine, McMaster University, Hamilton, ON Canada

**Keywords:** Antibody, Asthma, Cytokine, Immunoglobulins, Monoclonal

## Abstract

**Introduction:**

Immunoglobulins play a vital role in host immune response and in the pathogenesis of conditions like asthma. Therapeutic agents such as monoclonal antibodies target specific elements of the asthmatic inflammatory cascade. Decisions to utilize these medications are often based on systemic inflammatory profiling without direct insight into the airway inflammatory profile. We sought to investigate the relationship between immunoglobulin and cytokine profiles in the airway and systemic immune compartments of adult asthmatics.

**Methods:**

Blood sampling and bronchoscopy with bronchoalveolar lavage (BAL) were performed in 76 well-defined adult asthmatics. Antibody and cytokine profiles were measured in both BAL and serum using ELISA and quantibody arrays.

**Results:**

There was no relationship between BAL and serum levels of IgE. This is of significance in an asthma population. For some analytes, correlation analysis was significant (*P* < 0.05) indicating representativeness of our cohort and experimental setup in those cases. Nevertheless, the predictive power (*r*^2^) of the BAL-to-serum comparisons was mostly low except for TNF-α (*r*^2^ = 0.73) when assuming a simple (linear) relationship.

**Conclusion:**

This study highlights the importance of sample site when investigating the roles of immunoglobulins and cytokines in disease pathogenesis and suggests that both localized and systemic immune responses are at play. The prescription of asthma monoclonal therapy is generally based on systemic evaluation of cytokine and immunoglobulin levels. Our research suggests that this approach may not fully reflect the pathophysiology of the disease and may provide insight into why some patients respond to these targeted therapies while others do not.

**Supplementary Information:**

The online version contains supplementary material available at 10.1007/s00408-024-00699-x.

## Introduction

Asthma prevalence has seen a staggering rise with disease burden reaching epidemic proportions in the western world [1, 2]. 3–10% of asthmatics have severe asthma where symptoms remain poorly controlled despite optimal treatment with inhaled corticosteroids (ICS) and bronchodilators [3]. Many patients with severe asthma are treated with monoclonal antibodies which target the asthma inflammatory pathways, such as those targeting IgE, Interleukin (IL)-5 and its receptor, and the IL-4 receptor α subunit. These agents have demonstrated clinical efficacy in the treatment of severe asthma with type 2 inflammation [4–8].

It is well documented that the response to monoclonal treatment can vary from so-called “super-responders” to partial and non-responders. Understanding the reasons behind this varied response is complex with phenotypic characteristics, such as lower BMI, lower dose of maintenance oral steroids, and better baseline lung function, all predictors of a better response to treatment [5–8]. It has been suggested that how we assess suitability for monoclonal treatment, as well as response through serum biomarkers of disease may not be completely accurate. Certainly, it has been shown that 25% of asthma patients can have elevated sputum eosinophils without having corresponding elevated serum eosinophils [9]. Similarly, sputum rather than blood eosinophilia is a better predictor of response to mepolizumab and benralizumab, yet access to these medications is based on demonstrated blood eosinophilia [10]. Reliably predicting and assessing response to these agents is essential for better patient outcomes and cost effectiveness. As we continue to move to an era of personalized, targeted treatments this is of increasing relevance.

Immunoglobulins (Ig) play a vital role in the host immune response. Immunoglobulins are heterodimeric proteins consisting of two heavy chains and two light chains classified functionally into variable and constant domains [11]. Immunoglobulins function by acting as antigen cell surface receptors allowing for cell signaling and activation and also as soluble effector molecules which can neutralize potentially harmful antigens [11]. IgA, IgD, IgE, IgM and IgG are the main classes of the heavy chain constant domains, with IgG further divided into subclasses IgG1–IgG4 [11]. IgE has a role in hypersensitivity, allergy, and asthma pathogenesis, while IgA has an immune role at mucosal surfaces and IgG deficiency has been implicated in asthma exacerbations [11, 12]. Great advances have occurred with respect to understanding the role of immunoglobulins in immune homeostasis, enabling numerous diagnostic and therapeutic applications, including their use as monoclonal antibodies to treat asthma [11].

Research advancing the current understanding of the immunoglobulin profile within the bronchoalveolar lavage (BAL) and serum of asthmatic patients, the relationship between both and variation between individual asthmatics is of potential therapeutic relevance. Such research may offer real therapeutic insights into personalized targeted treatment options with monoclonal antibody therapy but also afford a possible explanation for treatment failures in asthmatics. We sought to gain further insight into the relationship between the immunoglobulin and cytokine profiles in the local airway and systemic immune compartments of adult asthmatic patients.

## Materials and Methods

### Patient Recruitment

The study was approved by the Clinical Research Ethics Committee of the Cork Teaching Hospitals. Informed written consent was obtained. 76 adult patients with a history of asthma (either documented change in FEV_1_ ≥ 12%/200 ml with bronchodilator or a positive methacholine or mannitol challenge) were recruited through a dedicated asthma clinic in a tertiary referral center [13]. Patients were scheduled for bronchoscopy, with spirometry, asthma control questionnaire-7 (ACQ-7), and blood sampling performed on the day. Spirometry was performed in accordance with ATS/ERS guidelines [14]. The cohort was stratified by both severity, as per GINA classification [mild (GINA 1 + 2), moderate (GINA 3), severe (GINA 4 + 5)], and asthma control as per ACQ-7 questionnaire [controlled (ACQ-7 < 1.5), uncontrolled (ACQ-7 ≥ 1.5)] [13, 15, 16] (Table 1). Patients underwent bronchoscopy in accordance with standard guidelines [17]. Bronchoalveolar lavage (BAL) was obtained as a standardized 180 ml (3 × 60 ml) sample from either the right middle lobe or lingula.

### Sample Processing

5 ml of neat BAL was aliquoted for cell differential analysis. The remaining BAL and blood were centrifuged at 500 g for 10 min at room temperature. The BAL supernatant was aliquoted, while the respective plasma (cytokine) and serum (immunoglobulin) was removed. All samples were stored at − 80 °C for cytokine and immunoglobulin analysis.

### Cell Differential Analysis

Neat BAL underwent cytocentrifugation, whereby 200 µl of BAL per microscope slide was centrifuged at 400 rpm for 2 min using a Shandon Cytospin 4 centrifuge (Thermo Fisher, Dublin, Ireland). Microscope slides were then differentially stained using a Kwik Diff commercial staining kit (Thermo Fisher, Dublin, Ireland) and microscopically analyzed to determine the BAL immune cell percentage counts.

### Immunoglobulins

Patient BAL and serum immunoglobulins IgM, IgA, IgD, IgG1, IgG2, IgG3, and IgG4 and BAL IgE were analyzed in accordance with inhouse procedures at RayBiotech using a Quantibody Human Ig Isotype Array 1 (Catalog#: QAH-ISO-1) (RayBiotech, Georgia, USA) which utilized sandwich ELISA-based technology, quantifiably measuring the eight human isotype immunoglobulins. BAL limits of detection (LOD): IgA (107-200,000pg/ml), IgD (343-50,000pg/ml), IgE (359-50,000pg/ml), IgM (134-100,000pg/ml), IgG1 (483-400,000), IgG2 (926-400,000pg/ml), IgG3 (23-50,000pg/ml), and IgG4 (22-16,667pg/ml) and serum IgA (13-20,0000pg/ml), IgD (94-50,000pg/ml), IgM (50-100,000pg/ml), IgG1 (80-400,000pg/ml), IgG2 (1951-

400,000pg/ml), IgG3 (112-50,000pg/ml), and IgG4 (120-50,000pg/ml). Serum IgE was measured using ImmunoCAP IgE assays (Thermo Fisher/Phadia, Uppsala, Sweden) in the hospital laboratory (LOD 2-5000kU/L).

### Cytokines

Interleukin (IL)-1β, IL-4, IL-5, IL-6, IL-8, IL-10, IL-13, IL-17, Interferon (IFN)-γ, and tumor necrosis factor (TNF)-α were measured in BAL and plasma using an electrochemiluminescence QuickPlex SQ 120 imager from Meso Scale Discovery (Gaithersburg, MD, USA).

### Statistical analyses

GraphPad Prism 9 software was used to perform all statistical analyses and to provide graphical representation of data. All data was assessed using normality and log-normality tests with the Shapiro–Wilk test applied to assess the normality of data. Data were considered statistically significant when *P* < 0.05 utilizing appropriate statistical tests (Mann–Whitney T test, Spearman’s R) as required. Pearson’s correlation and linear regression was performed to assess relationships between variables.

## Results

**Table 1:** Patient demographics and clinical characteristics

		Mild (GINA 1–2)	Moderate (GINA 3)	Severe (GINA 4–5)
Patients	*n* = 76 (%)	26 (34%)	17 (22%)	33 (44%)
*Female*		16 (62%)	12 (71%)	20 (61%)
Age (years)	[Mean (SD)]	[46.9 (15.1)]	[52.6 (14.3)]	[51 (12.7)]
	[Median (IQR)]	{49 (33–60)]	[57 (39–650]	[51 (40.5–73)]
Smoking status	Current (%) Ex-smoker (%) Never smoker (%)	3 (12%) 6 (23%) 17 (65%)	1 (6%) 4 (24%) 12 (71%)	3 (9%) 11 (33%) 19 (58%)
Asthma control questionnaire-7 (ACQ-7)	[Mean (SD)]	[1.1 (0.6)]	[1.9 (1.0)]	[3.2 (1.1)]
	[Median (IQR)]	[1.1 (0.6–1.6)]	[1.9 (1.1–2.6)]	[3.1 (2.5–4.1)]
ACQ-7 < 1.5	*n* = 28 (37%)	*n* = 19 (25%)	*n* = 7 (9%)	*n* = 2 (3%)
ACQ-7 > 1.5	*n* = 48 (63%)	*n* = 7 (9%)	*n* = 10 (13%)	*n* = 31 (41%)
Inhaled corticosteroids (ICS) (FDP 250) (*n* = 65)	[Mean (SD)]	[442.3 (470.7)]	[926.5 (521.2)]	[1038 (464.0)]
	[Median (IQR)]	[375 (0-1000)]	[1000 (500–1000)]	[1000 (750–1250)]
IgE (kU/L)	[Mean (SD)]	[361.3 (643.2)]	[168.8 (237.8)]	[270.6 (436.3)]
	[Median (IQR)]	[96.1 (30.7–399.0)]	[138.0 (63.1–180.0)]	[100.1 (22.4-286.3)]
FEV_1_ (%Predicted)	[Mean (SD)]	[86.9 (13.1)]	[86.7 (20.7)]	[64.9 (22.7)]
	[Median (IQR)]	[88.0 (78.8–96.0)]	[81.6 (74.0-101.5)]	[67.0 (46.5–80.5)]
FeNO (ppb)	Readings from	23	14	24
	[Mean (SD)]	[30.5 (27.4)]	[22.7 (11.4)]	[20.2 (25.1)]
	[Median (IQR)]	[21.0 (11.0–43.0)	[21.0 (15.5–32.0)]	[13.0 (19.3–23.8)]
BMI (kg/m^2^)	[Mean (SD)]	[26.0 (4.5)]	[27.5 (6.9)]	[30.0 (6.5)]
	[Median (IQR)]	[24.5 (22.4–30.0)]	[26.0 (22.5–31.2)]	[29.0 (25.1–33.8)]
Blood Eosinophil count (10^9^/L) *n* = 73	[Mean (SD)]	[0.3 (0.2)]	[0.3 (0.4)]	[0.3 (0.3)]
	[Median (IQR)]	[0.2 (0.1–0.3)]	[0.2 (0.1–0.4)]	[0.2 (0.1–0.3)]

This table highlights key patient demographics and clinical results obtained on the day of bronchoscopy

Descriptive statistics were used to calculate the mean, standard deviation, median and interquartile range

*ACQ-7* Asthma Control Questionnaire, *ICS* Inhaled Corticosteroid, *FEV1* Forced Expiratory Volume in the first second, *FeNO* Fractional exhaled Nitric Oxide, *BMI* Body Mass Index, *IQR* Interquartile range

### Patient Demographics

The patient cohort consisted of 76 adult asthma patients in which total antibody immunoglobulin and cytokine levels were measured in BAL and blood. The cohort was stratified by severity (GINA) (34% mild (GINA 1 + 2), 22% moderate (GINA 3), and 44% severe (GINA 4 + 5) asthma) and level of asthma control (ACQ-7) (37% controlled (ACQ-7 < 1.5) and 63% were uncontrolled (ACQ-7 ≥ 1.5)). Those with severe asthma had overall poorer clinical parameters with a higher ACQ-7 score and lower FEV_1_. Nine patients, all of whom were in the severe asthma group (GINA 4 + 5) were on long-term, maintenance oral corticosteroids (Range 5–30 mg), while 14.5% of asthmatics were ICS naïve at time of bronchoscopy (Table 1). Of those who were ICS naïve, all were classed as GINA 1 or 2, and 45.5% were considered uncontrolled as per their ACQ-7 score. No patient was on monoclonal therapy at the time of sampling.

### BAL and Serum Immunoglobulins

**Fig. 1:**
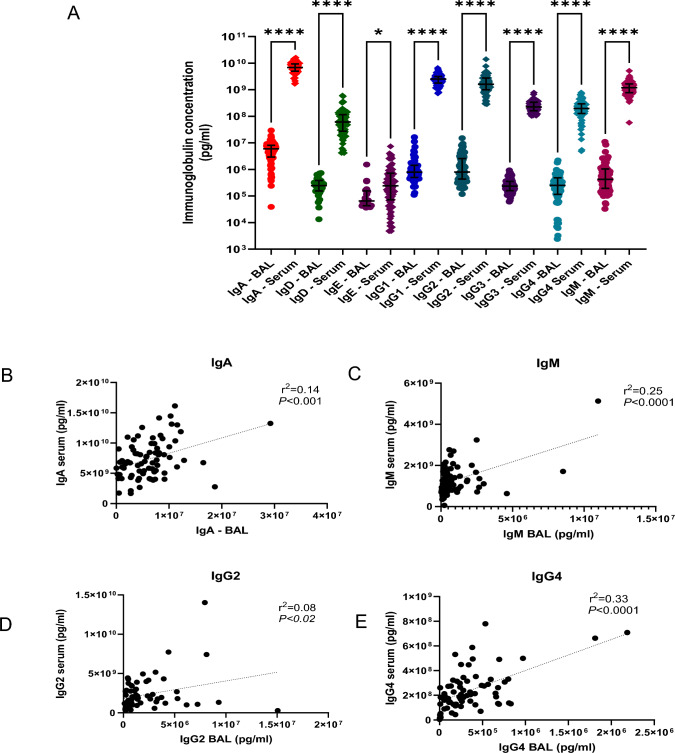
BAL and serum immunoglobulins. IgA, IgD, IgM, IgG1, IgG2, IgG3, and IgG4 (*P* < 0.0001) and IgE (*P* < 0.01) were found to have a significantly greater concentration in serum versus BAL fluid (**A**). Correlations were noted between serum IgA and BAL IgA (r^2^ = 0.14, *P* < 0.001) (**B**), serum IgM and BAL IgM (r^2^ = 0.25, *P* < 0.0001) (**C**), serum IgG2 and BAL IgG2 (*r*^2^ = 0.08, *P* < 0.02) (**D**), and serum IgG4 and BAL IgG4 (*r*^2^ = 0.33, *P* < 0.0001) (**E**) but not for IgD, IgE, IgG1, or IgG3 (not shown). Although, these correlations were statistically significant they were weak correlations as per the *r*^2^ value. *N* = 76. Statistical tests employed included Mann–Whitney, Pearson correlation, and linear regression analysis

Serum and BAL immunoglobulin levels were significantly different between sites with increased levels of IgM, IgA, IgD, IgG1, IgG2, IgG3, IgG4 (*P* < 0.0001), and IgE (*P* < 0.01) in serum versus BAL samples (Fig. 1A). This may in part be due to the natural dilution effect of BAL sampling. Several correlations were observed to be significant (*P* < 0.05) when serum and BAL levels of some of the analytes were compared; serum and BAL IgM (*r*^2^ = 0.25, *P* < 0.0001), IgA (r^2^ = 0.14, *P* < 0.001), IgG2 (r^2^ = 0.08, *P* < 0.02), and IgG4 (r^2^ = 0.33, *P* < 0.0001) (Fig. 1B–E). Overall, however, these relationships were mostly weak as denoted by a r^2^ value of < 0.65 [18]. IgD, IgE, IgG1, and IgG3 serum and BAL levels did not correlate. Both BAL and serum immunoglobulin levels were analyzed for a possible relationship with asthma control (ACQ-7) and severity (GINA), but no significant relationship was observed for either.

### Immunoglobulin Levels Reflect Local Airway and Systemic Cytokine Signals

**Fig. 2:**
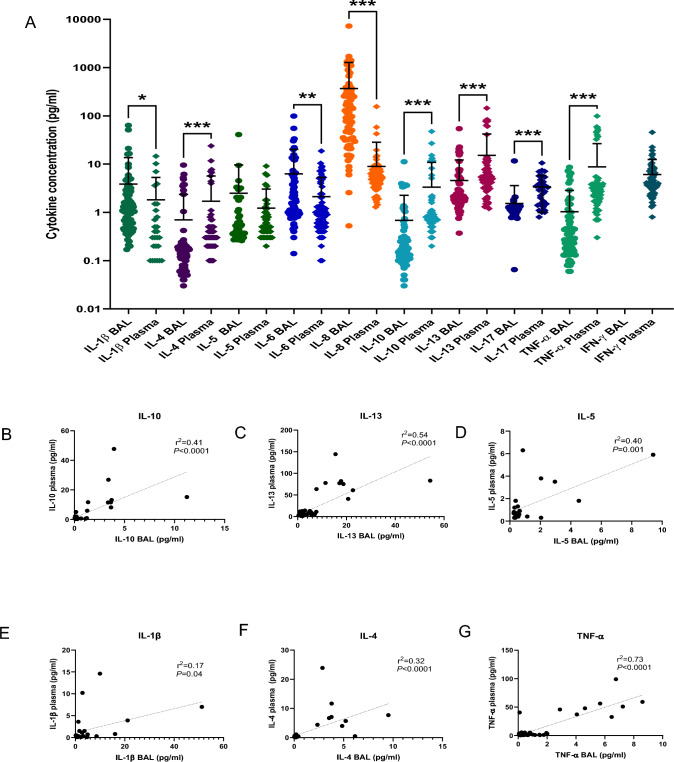
BAL and plasma Cytokines. IL-8 (*P* < 0.0001), IL-1β (*P* = 0.002), and IL-6 (*P* = 0.0002) were present in greater quantities in BAL, while IL-17, IL-10, IL-13, IL-4, and TNF-α were significantly greater in plasma (*P* < 0.0001) (**A**). No difference was noted for IL-5. Correlations were found between serum and BAL for some cytokines, including IL-10 (*r*^2^ = 0.41, *P* < 0.0001) (**B**), IL-13 (*r*^2^ = 0.54, *P* < 0.0001) (**C**), IL-5 (*r*^2^ = 04, *P* = 0.001) (**D**), IL-1β (*r*^2^ = 0.17, *P* = 0.04) (**E**), IL-4 (*r*^2^ = 0.32, *P* < 0.0001) (**F**), TNF-α (*r*^2^ = 0.73, *P* < 0.0001) (**G**), and IL-6 (*r*^2^ = 0.14, *P* = 0.002) (not shown). Although these relationships were statistically significant, with the exception of TNF-α which demonstrates a strong correlation, all other relationships were weak. A strong relationship was considered to be one where *r*^2^ > 0.65. *N* = 76. Statistical tests included Mann–Whitney, Pearson's correlation, and linear regression analysis

Plasma and BAL cytokines were found to significantly differ between sites with IL-8 (*P* < 0.0001), IL-1β (*P* = 0.002), and IL-6 levels *(P* = 0.0002) greater in BAL airway samples, while IL-17, IL-10, IL-13, IL-4, and TNFα levels *(P* < 0.0001) were greater in plasma (Fig. 2A). IL-5 levels were not significantly different between BAL and plasma samples. When the relationship between plasma and BAL cytokines was explored, although there were significant relationships between some analytes (*P* < 0.05), these were found to be mostly weak associations with the exception of TNF-α. These cytokines included plasma and BAL IL-10(*r*^2^ = 0.41, *P* < 0.0001), IL-1β (*r*^2^ = 0.17, *P* = 0.04), IL-4 (*r*^2^ = 0.32, *P <* 0.0001), IL-6 (*r*^2^ = 0.14, *P* = 0.002), IL-13 (*r*^2^ = 0.54, *P <* 0.0001), IL-5 (*r*^2^ = 0.4, *P* = 0.001), and TNFα (*r*^2^ = 0.73, *P* < 0.0001) (Fig. 2B–G). There was no significant correlation observed for IL-8 or IL-17.

Correlations were identified between serum immunoglobulins and plasma cytokines and within BAL levels of immunoglobulins and cytokines, but correlations did not exist within both compartments. Again, although these correlations were statistically significant in terms of p value, the strength of most of the relationship was weak. Some of these correlations are highlighted in Supplementary Figures S1 and S2.

## Discussion

Research which advances the current understanding of the immunoglobulin and inflammatory profile within the BAL and serum of asthmatic patients, in particular, highlighting differences between local airway and systemic profiles, may offer real potential therapeutic insights. We therefore sought to interrogate and compare the immune response localized to the asthmatic airway with that observed systemically, with a particular reference to immunoglobulin profiling.

Overall, absolute immunoglobulin levels were higher in serum. This was not unanticipated and is potentially accounted for by the dilution effect which occurs as part of the nature of the BAL sampling process. However, several cytokine levels were higher in BAL samples (IL1β, IL-6, IL-8), while others were higher in plasma (IL-4, IL-13, IL-17, IL-10, and TNFα); therefore, the BAL dilution effect is not wholly responsible for varying levels. Instead, this probably represents the function of the mucosal, epithelia rich, airway site, and the natural distribution of immunoglobulins. Our analysis detected 28% of IgE in BAL, while most other immunoglobulins were detected at 100%. We propose that this is not just due to dilution alone. To further investigate why 28% of patients had detectable IgE signals and the rest did not, we compared the groups and found that there was a significant difference in FEV_1_ values with a lower % predicted in those with a detectable IgE (mean 80.6% v 68.5%, *P* = 0.03), there were more males and a greater proportion of ex-smokers in the IgE-detected group (52% v 18%). Therefore, the detection of IgE may reflect a more inflammatory clinical phenotype. Furthermore, in the IgE-detected group, there was a correlation between %eosinophils in BAL and serum eosinophils (*P* = 0.001, *r* = 0.69) and plasma IL-5 (*P* = 0.01, *r* = 0.55) levels which may further reflect this.

Our study demonstrated weak relationships between BAL and serum levels of IgM, IgA, IgG2, and IgG4,(Fig. 1B–E), while no correlation was observed between airway and systemic levels of IgD, IgE, IgG1, and IgG3, suggesting systemic and local airway immunoglobulin levels are not fully in concordance. Furthermore, with the exception of TNF-α, weak relationships were also found between BAL and plasma levels of IL-10, IL-1β, IL-4, IL-5, IL-6, and IL-13 (Fig. 2B–G). Overall, the best predictor of the BAL analyte level was serum TNF-α with a coefficient of determination of 0.73 meaning that 73% of TNF-α BAL levels can be deduced from its serum levels. The same could not be said for any of the other analytes examined in this study. Treatment decisions, therefore, should be made with great caution when only serum immunoglobulin and cytokine levels other than those of TNF-α are available, as systemic and BAL levels of both immunoglobins and cytokines are not fully reflective of each other and measurements in one compartment may not be an accurate representation of the pathophysiology occurring in the other.

IgE is a potent monomeric immunoglobulin associated with hypersensitivity and allergic asthma and is the most clinically relevant of the immunoglobulins analyzed in our study [11]. IgE is a target of omalizumab, a humanized anti-IgE monoclonal antibody used to treat severe asthma [5]. The prescription of omalizumab is based on serum IgE levels which our data suggest may not be reflective of the airway inflammatory profile. Studies which have compared induced sputum levels of IgE to that of serum IgE have shown contradictory reports with some showing a correlation between the two levels, while others have shown no correlation between the two compartments [19–22]. Furthermore, studies which use more invasive measures of airway sampling would suggest that IgE levels are higher in the airways which may indicate localized production of IgE not reflected in the serum [23]. Therefore, the use of serum IgE as a marker for commencing biological therapy in severe asthma may not be appropriate. In fact, these discrepancies may explain why some patients meeting the current clinical criteria for treatment with omalizumab therapy fail to display a beneficial clinical response [24–26].

Our study highlights immunoglobulin variation is not only present between different individuals but also within individuals, with variation in immunoglobulin levels existing within the airway and systemically. The lack of an observed relationship between airway and systemic IgE is of clinical relevance to asthma, having possible implications for drug efficacy within an asthmatic cohort. As mentioned, important therapeutic interventions with omalizumab are made based on systemic rather than airway immunoglobulin profiles. The response to this therapy is not universally positive. This suggests that such an approach for anti-IgE monoclonal therapy may not be optimal in all cases and that perhaps further defining the airway inflammatory profile may afford a more sophisticated, targeted, individualized approach to patient treatment with such agents. We believe that our findings may partly explain the clinical variation in response to omalizumab and given the costs associated with such treatment in ‘non-responders’ may justify further individual patient profiling prior to prescription of these medications. We believe that studies assessing clinical response based on airway profiling, in addition to the current systemic profiling are now warranted.

### Advantages and Limitations

There are some potential limitations to our study. The lack of correlation between serum and BAL IgE could possibly be because IgE was analyzed using an ImmunoCAP system while the other immunoglobulins were analyzed with sandwich ELISA which slightly vary in target detection. Furthermore, only 28% of samples had detectable BAL IgE levels compared with nearly 100% detection for other immunoglobulins, but we did explore this further and believe it may reflect a group of patients with elevated inflammation within the airways. Dilution because of BAL sampling may also have affected results when compared to serum levels of immunoglobulins, but this was not the case with cytokine levels. However, our study has several strengths. We have a large patient number of clinically well-defined asthma patients. Furthermore, both BAL and blood samples were acquired on the same day and analyzed in a similar manner to minimize potential confounders. We acknowledge that bronchoscopy may not be viable in all centers or in all patients who are being screened prior to starting monoclonal therapy; however, studies on the relationship between sputum and blood IgE have been contradictory [19–22]. Bronchoscopy offers an advantage in that it allows for visual inspection and guarantees sample acquisition to fully phenotype patients prior to starting monoclonals.

## Conclusion

Overall, our study highlights the importance of sample site when measuring and investigating immunoglobulins and inflammation and their potential pathophysiological roles in airway diseases, such as asthma. The decision to utilize monoclonal therapy in asthma is generally taken based on systemic evaluation of cytokine and immunoglobulin levels without clear sight of how such measurements relate to the local airway levels. Our research suggests that this approach may not always accurately reflect the processes driving the pathophysiology relevant to the disease, especially when extrapolating possible relationships to individual patients. Furthermore, our observations may explain why some patients demonstrate a positive response to these targeted therapies, while others do not.

### Supplementary Information

Below is the link to the electronic supplementary material.

Supplementary material 1 (DOCX 469.5 kb)
