# Supplementary material for: Airway and Systemic Immunoglobulin Profiling and Immune Response in Adult Asthma
Source: Lung. 2024 May 7;202(3):281–9. doi: 10.1007/s00408-024-00699-x (PMC11142944; doi:10.1007/s00408-024-00699-x)
Supplement: Supplementary file 1 — Supplementary material 1 (DOCX 469.5 kb) [file 408_2024_699_MOESM1_ESM.docx]

**Airway and systemic immunoglobulin profiling and immune response in adult asthma**

Laura J. Walsh^a, b, c^† Ashley Sullivan^a, d, e,^ †, Chris Ward^f^, Eoin B. Hunt^b, c^, Susan Lapthorne^d^, Joseph A. Eustace^c^, Liam J Fanning^a, c^, Barry J. Plant^b, c^, Paul M. O’Byrne^g^, John A. MacSharry^a, d, e*^, Desmond M. Murphy^b, c*^

*Ashley Sullivan and Laura J. Walsh should be considered joint first authors.*

*John A. MacSharry and Desmond M. Murphy should be considered joint senior authors.*

***Affiliations:*** *^a^The School of Medicine, University College Cork, Cork, Ireland.*

*^b^The Department of Respiratory Medicine, Cork University Hospital, Cork, Ireland.*

*^c^The HRB funded Clinical Research Facility, University College Cork, Cork, Ireland.*

*^d^APC Microbiome Ireland, University College Cork, Cork, Ireland.*

*^e^The School of Microbiology, University College Cork, Cork, Ireland.*

*^f^Translational and Clinical Research Institute Cellular Medicine, Newcastle University, Newcastle upon Tyne, UK.*

*^g^The Michael G DeGroote School of Medicine, McMaster University, Hamilton, Ontario, Canada.*

*†= Joint 1^st^ Authors. *= Joint Senior Authors*

**Corresponding author:**

Prof Desmond Murphy

The Department of Respiratory Medicine,

Cork University Hospital,

Cork, Ireland

Email: [desmond.murphy@hse.ie](about:blank)

Phone number: 021-4234154

**Supplementary Material**

| Immunoglobulin (Ig)N=76 | Detectable Levels (%) | Median (IQR- interquartile range) | *P* value when BAL and plasma levels were compared |
| --- | --- | --- | --- |
| IgA - BAL (pg/ml) | 76/76 (100%) | 6.0 x10^6^ (2.9x10^6^-8.1 x10^6)^ | *P* <0.0001 |
| IgA - Plasma (pg/ml) | 76/76 (100%) | 6.9x10^9^ (5.0 x 10^9^- 9.3 x10^9^) |  |
| IgD - BAL (pg/ml) | 28/76 (37%) | 2.4 x10^5^ (1.6 x10^5^-3.8x 10^5^) | *P* <0.0001 |
| IgD – Plasma (pg/ml) | 74/76 (96%) | 6.1x10^7^ (2.8 x 10^7^-1.2 x 10^8^) |  |
| IgE – BAL (pg/ml) | 21/76 (28%) | 6.4 x 10^4^ (4.3 x10^4^-1.6 x 10^5^) | *P* <0.01 |
| IgE – Plasma (pg/ml) | 75/75 (100%) | 2.4 x 10^5^ (7.2 x10^4^ – 7.2 x 10^5^) |  |
| IgG1 – BAL (pg/ml) | 65/76 (86%) | 7.9x 10^5^ (5 x 10^5^ – 1.4 x 10^6^) | *P* <0.0001 |
| IgG1 – Plasma (pg/ml) | 76/76 (100%) | 2.5 x 10^9^ (1.8 x10^9^-3.2 x 10^9^) |  |
| IgG2 – BAL (pg/ml) | 72/76 (96%) | 8.0 x10^5^ (4.3 x 10^5^-2.6 x 10^6^) | *P*< 0.0001 |
| IgG2 – Plasma (pg/ml) | 76/76 (100%) | 1.6 x10^9^ (1.0x10^9^ -2.8 x10^9^) |  |
| IgG3 – BAL (pg/ml) | 76/76 (100%) | 2.3 x 10^5^ (1.6x 10^5^-3.6 x 10^5^) | *P* <0.0001 |
| IgG3 – Plasma (pg/ml) | 76/76 (100%) | 2.3 x 10^8^ (1.6 x 10^8^-3.4 x 10^8^) |  |
| IgG4 – BAL (pg/ml) | 73/76 (96%) | 2.5 x 10^5^ (1.2 x 10^5^-4.9 x 10^5^) | *P* <0.0001 |
| IgG4 – Plasma (pg/ml) | 76/76 (100%) | 2.0 x 10^8^ (1.3 x 10^8^- 3.0 x 10^8^) |  |
| IgM – BAL (pg/ml) | 76/76 (100%) | 4.2 x10^5^ (1.9 x10^5^- 1.0 x 10^6^) | *P* <0.0001 |
| IgM – Plasma (pg/ml) | 76/76 (100%) | 1.2x 10^9^ (7.8 x 10^8^-1.7 x 10^9^) |  |

**Supplementary Table 1: Patient BAL and plasma immunoglobulin levels.** This table depicts the percentage of patients displaying detectable levels of the immunoglobulins IgA, IgD, IgE, IgG1, IgG2, IgG3, IgG4 and IgM with the overall [Median (IQR)] levels detected (pg/ml). The significant difference between each immunoglobulin when levels in the plasma and BAL were compared are also highlighted. Statistical tests: Descriptive statistics and Mann-Whitney.

C

**Supplementary Figure S1: Local and systemic antibody and cytokine correlations.** Antibody and cytokine concentrations in patient BAL and blood were analyzed. Some of the key results are highlighted here. BAL IgD levels negatively correlated with BAL IL-17 levels (r^2^=0.67, *P*=0.01) (A). This can be considered a strong relationship. A strong relationship was considered to be one where r^2^>0.65. Serum IgD levels did not correlate with plasma IL-17 levels (B). Serum IgA negatively correlated, weakly, with plasma IL-13 (r^2^=0.07, *P*=0.03) (C) but BAL IgA did not correlate with BAL IL-13 levels (D). BAL IgM correlated weakly, with BAL IL-6 (r^2^=0.08, *P*=0.02) (E) but serum IgM levels did not correlate with plasma IL-6 levels (F). N=76. Statistical tests used: Pearson’s test and linear regression.

**Supplementary Figure S2: Local and systemic antibody and cytokine correlations.** Antibody and cytokine concentrations in patient BAL and blood were analyzed. Serum IgA levels negatively correlated with plasma IL-10 levels (r^2^=0.07, *P*=0.04) (A) but BAL IgA levels did not correlate with BAL IL-10 levels (B). Serum IgA negatively correlated with plasma TNF-α levels (r^2^=0.05, *P*=0.049) but the same correlation was not seen in the blood (D). Serum IgG4 correlated with plasma IL-17 (r^2^=0.14, *P*=0.04*)* but the same correlation was not seen in the BAL fluid. Although, the correlations were statistically significant in terms of their p value the strength of the relationships were very weak. A strong relationship was considered to be one where the r^2^ >0.65. N=76. Statistical tests used: Pearson’s test and linear regression.
